# Quyushengxin Formula Causes Differences in Bacterial and Phage Composition in Ulcerative Colitis Patients

**DOI:** 10.1155/2020/5859023

**Published:** 2020-05-11

**Authors:** Haojie Yang, Dan Gan, Ying Li, Xiaosu Wang, Lei Jin, Kaijian Qin, Can Cui, Jiong Wu, Zhenyi Wang

**Affiliations:** ^1^Department of Colo-Proctology, Yueyang Hospital of Integrated Traditional Chinese and Western Medicine, Shanghai University of Traditional Chinese Medicine, Shanghai 200437, China; ^2^Department of Gastroenterology, Yueyang Hospital of Integrated Traditional Chinese and Western Medicine, Shanghai University of Traditional Chinese Medicine, Shanghai 200437, China

## Abstract

**Background:**

Ulcerative colitis (UC) is a chronic inflammatory disease that affects the colon and the rectum. Recently, some studies have shown that microorganisms in the gut play important roles in many chronic diseases such as UC.

**Methods:**

To study the candidate viruses and bacteria involved in UC and to investigate the therapeutic mechanism of Quyushengxin formula (QYSX) in UC patients, metagenomic sequencing was performed on the feces from healthy donors and UC patients before and after QYSX treatment.

**Results:**

QYSX improved the symptoms of UC. In all participants, *Caudovirales* and *Herpesvirales* were the most dominant viruses. The abundance of *Caudovirales* in UC patients was significantly higher than that in the normal controls, while QYSX restored *Caudovirales* abundance. Furthermore, the abundance of *crAssphage* was enhanced in UC patients compared with the normal control, while the diversity was then decreased after QYSX treatment. However, there was no significant difference (*P* > 0.05). Additionally, other non-*crAssphage* bacteriophages including *phiST*, *SP-10*, and *phi17:2* were higher in UC patients and QYSX decreased these viruses, while the trends of *MED4−213*, *P-HM1*, and *P−HM2* were adverse. Interestingly, *PhiDP23.1* was only found in UC patients before and after QYSX treatment. In addition, *Bifidobacterium*, *Bacteroidetes*, *Prevotellaceae*, *Actinobacteria*, and *Corynebacteriales* were the biomarkers in UC patients after QYSX treatment due to their high abundance. GO terms and KEGG analysis showed that the identified gut microbiome was involved in many biological processes and pathways.

**Conclusions:**

QYSX could regulate disordered gut microbiome and phages, indicating that QYSX has great therapeutic potential for UC.

## 1. Introduction

Ulcerative colitis (UC), one of the inflammatory bowel diseases (IBDs), is a chronic inflammatory disease affecting the colon and the rectum. The peak incidences of UC occur between 30 and 40 years of age. UC is characterized with rectal bleeding, diarrhea, dystonia, and sometimes with low abdominal pain [1]. The severity of colitis affects the occurrence and development of colorectal cancer and leads to intestinal dysfunction. A recent study has shown that the pathogenesis of UC is multifactorial, including genetic susceptibility, epithelial barrier defects, dysregulated immune responses, environmental factors, and intestinal disorders [2]. Currently, the diagnosis of UC is mainly based on a combination of clinical symptoms, endoscopic findings, and histology [3], and the main treatments for UC are some drugs such as 5-aminosalicylic acid, glucocorticoids, and immunosuppressor. However, these drugs are not suitable for long-term treatment owing to their side effects, including diarrhea, abdominal pain, nausea, and vomiting. Therefore, there is an urgent need to develop natural and safe medications for UC.

Increasing evidence showed that the traditional Chinese medicines can be applied to treat UC [4]. Feng et al. [5] indicated that Shenzhu Capsule contained two herbal medicines (Renshen and Baizhu) and improved UC by altering TNF signalling pathway, Toll-like receptor signalling pathway and NF-*k*b signalling pathway. Qingchang Wenzhong Decoction comprising of eight kinds of herbs attenuated dextran sulphate sodium- (DSS-) induced UC in rats by upregulating macrophage-stimulating protein concentration/receptor tyrosine kinase signalling pathway [6]. In addition, Quyushengxin formula (QYSX), known as Kuijie formula, contains eight kinds of herbs. QYSX is the prescription widely used for UC treatment in China, due to its antibacterial, anti-inflammatory, and antidiarrheal properties. A study by Wu et al. showed that QYSX administration significantly improved the disease activity index and colonic mucosa damage index in UC by decreasing the level of IL-1*β* and increasing the expression of IL-10 in rat model [7]. Furthermore, another study demonstrated that after QYSX combination treatment, the treatment efficiency of UC significantly improved based on the results of clinical practice [8]. Despite the therapeutic effect of QYSX on UC, its potential mechanism is still unknown.

The intestinal flora in humans comprises a complex ecosystem, which mainly includes bacteria (more than 10^14^) and other microorganisms, such as fungi, parasites, viruses, and Archaea [9]. In recent years, accumulating studies suggest that the intestinal microbiome plays a critical role in the occurrence, development, and prognosis of UC [10, 11]. In IBD patients, the biodiversity including *α*-diversity of fecal microbiome is usually reduced compared to the healthy controls [12]. In addition, some studies have demonstrated that dysbiosis occurs in IBD [13] and displays the decreased abundance of *Firmicutes*, *Bacteroides*, and *Lachnospiraceae* and an increase in *Gammaproteobacteria* [14–16]. Another study by Paramsothy et al. [17] showed that fecal microbiota transplantation (FMT) effectively alleviated UC via increasing microbial diversity and altering microbial composition (*Sutterella wadsworthensis*, *Fusobacterium gonidiaformans*, and *Escherichia*). For the virome, the bacteriophages temperate dsDNA *Caudovirales* and ssDNA *Microviridae* are predominant in healthy gut [9, 15]. In Crohn's disease and UC patients from United Kingdom and America, virome richness increased [9, 18]. Although numerous attentions have been given to gut microbiome, the effects of QYSX on the function and composition of intestinal microorganisms are still unknown.

In this study, metagenomic sequencing was performed on feces from healthy volunteers and UC patients before and after QYSX treatment. The metagenomic markers associated with UC and the effects of QYSX on the function and structure of intestinal microorganism were identified. The results of this study will provide significant basis for the application of QYSX in the treatment of UC.

## 2. Materials and Methods

### 2.1. Study Design and Subjects

Eight healthy controls and eight UC patients were recruited in this study between 1 July 2017 and 30 May 2018 in the Department of Coloproctology, Yueyang Hospital of Integrated Traditional Chinese and Western Medicine. Fecal samples were collected from healthy controls and UC patients before and after QYSX treatment. The control groups included samples from healthy donors and the samples from patients before treatment. The healthy donors received no treatment. Two colonoscopies were performed in UC patients before and after treatment. The information of the participants is shown in Table S1.

The inclusion criteria for UC patients were as follows: (i) patients who met the diagnostic criteria of Western Medicine and have had this disease for more than four weeks; (ii) male and female patients between the age range of 16 and 65 years; and (iii) all volunteered participants that all joined the trial and signed an informed consent form. The exclusion criteria for UC patients were as follows: (i) pregnancy or drug allergies; (ii) serious diseases related to cardiovascular system, liver, and kidney, or mental disorders; (iii) intestinal stenosis, colon cancer, and related diseases; (iv) diagnosis of Crohn's disease or autoimmune diseases; (v) noncompliance with prescribed medications or treatment; and (vi) participation in other clinical trials in the last 3 months.

Healthy controls were enrolled based on the following criteria: (i) good health condition, regular work schedule, no past medical history of UC, no food and drink preferences, and absence of all kinds of bad hobbies; (ii) regular and normal bowel movements; (iii) no antibiotic use or continuous intake of probiotics and yogurt in the past 3 months; and (iv) not involved in other clinical studies in the past 3 months. Those who had the following conditions were excluded from the study: (i) a state of disease and (ii) pregnant or lactating individuals.

This study was approved by the Ethics Committee of Yueyang Hospital of Integrated Traditional Chinese and Western Medicine, Shanghai University of Traditional Chinese Medicine. All participants provided written informed consent. Additionally, we confirm that this study adhered to CONSORT guidelines This trial is registered with Current Controlled Trials ChiCTR1900023349.

### 2.2. Treatment

The composition of QYSX formula was 45 g of *Astragalus membranaceus* (Fisch.) Bunge, 30 g of *Pseudostellaria heterophylla* (Miq.) Pax, 15 g of *Atractylodes macrocephala* Koidz., *Semen persicae*, *Ligusticum chuanxiong* Hort, and *Rehmannia glutinosa* Libosch, and 20 g of *Euphorbia humifusa* Willd and *Kummerowia striata* (Thunb.) Schindl. The specimens of these eight kinds of herbs were identified and stored in a publicly available herbarium of Shanghai University of Traditional Chinese Medicine. This was obtained from the preparation room of Yueyang Hospital. Each bag of QYSX was 200 ml, and one bag was taken each time and two times per day. QYSX was taken two hours after breakfast and dinner. The treatment process lasted for 12 weeks. The full protocol is available with the corresponding author and can be obtained on reasonable request.

### 2.3. Specimen Collection

Fresh feces of mung bean size from participants was collected using a sterile culture tube in the morning. Samples were stored with ice bag and sent to proctology department of the hospital within six hours and stored at −20°C. Stool samples were sent to the laboratory using dry ice to maintain freezing temperature and were transferred to the refrigerator at −80°C for future use.

### 2.4. DNA Extraction and Metagenomic Sequencing

According to the manufacturer's instructions, total DNA was extracted from 180.0 to 220.0 mg of homogenized fresh fecal sample using TIANamp Stool DNA Kit (Tiangen Biotech (Beijing) Co., Ltd., Beijing, China). The concentration and purity of DNA were detected by agarose gel electrophoresis and Nanodrop. DNA was interrupted and fragmented by Covaris, and library was constructed using NEBNext® UltraTM DNA Library Prep Kit according to the manufacturer's instructions. After end repair and adaptor ligation, 300 bp adaptor-ligated DNA were selected and cleaned up using AMPure XP beads (Beckman Coulter). All samples were then sequenced using the Illumina platform with HiSeq 2500 instrument.

### 2.5. Data Processing

The original sequencing data were evaluated with FastQC and were filtered using Trimmomatic to obtain relatively accurate and effective data. IDBA_UD was used to assemble clean reads into long contigs. Contigs were obtained based on the overlap relationship between reads. The optimal Kmer assembly results were selected by comprehensively evaluating the assembly results of multiple Kmer. ORF from the splicing results was predicted using Prodigal, and genes with length of 100 bp were selected and translated into amino acid sequences. In order to obtain nonredundant gene sets, CD-HIT software was used to remove the redundancy of gene prediction results. Clean reads of each sample were aligned to the sequences of nonredundant gene sets using Bowtie 2 and the aligned reads were obtained using SAM tools. The virus data were blasted with the database of NCBI nonredundant protein sequences (NR). In addition, Shannon–Wiener index and Simpson index were used to characterize the diversity of microbial gene. According to the microbial taxonomic information database of NCBI (http://ncbi.nlm.nih.gov/), the taxonomic annotation information of genetic species was obtained. The abundance level of species was counted in taxonomic levels of kingdom, phylum, class, order, family, genus, and species. To compare the protein sequence with KEGG database (http://www.kegg.jp), the KO number corresponding to the sequence was obtained using GhostKOALA. GO terms were obtained according to UniProt ID and DIAMOND is used to compare the protein sequence with the UniProt database (http://www.uniprot.org/). Screening conditions were *e*-value < 1*e* − 5 and score > 60. The abundance of GO functional levels in each sample was calculated.

## 3. Results

### 3.1. Effect of QYSX on UC Patients

Eight healthy donors and eight UC patients were recruited in this study. Endoscopy was performed on the UC patients before and after QYSX treatment, respectively. Before QYSX treatment, patients with mild UC showed mucosa with the absence of vascular pattern, oedema, redness, and superficial ulceration, while they showed mucosa with mild oedema and redness after QYSX treatment (Figure S1). For patients with moderate UC, endoscopy displayed mucosa with the absence of vascular pattern and friability, oedema, redness, and the spontaneous bleeding mucosa with array of aphthous ulcers. However, after QYSX treatment, mucosal hyperemia and oedema were significantly reduced and no obvious ulcer was observed.

### 3.2. Effects of QYSX on Virome Diversity in UC Patients

**Figure 1:**
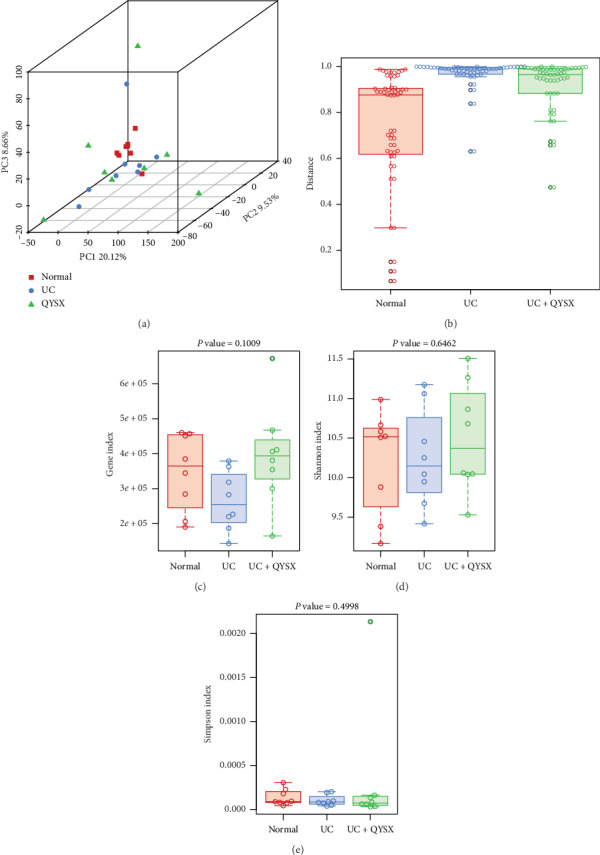
Effects of QYSX on fecal virome diversity. (a) Principal component analysis (PCA) of virome in healthy donors and ulcerative colitis (UC) patients before and after QYSX treatment. (b) Box plot analysis of the distance among all the samples. (c) Virome diversity analyzed by gene index. (d) Virome diversity analyzed by Shannon index analysis. (e) Virome diversity analyzed by Simpson index analysis. Normal: healthy donors; UC: UC patients; UC + QYSX: UC patients after QYSX treatment. QYSX alters virome composition in patients.

Beta-diversity was analyzed using principal component analysis (PCA) to study the relationship among the fecal microbiota in different groups at the same species level (Figure 1(a)). PCA showed that the virome in UC patients before QYSX treatment was different from that in healthy donors. However, QYSX significantly decreased the differences in virome between UC patients and healthy controls. Furthermore, the box plot of the distance in UC patients was higher than that in healthy controls, which showed a difference between healthy controls and UC patients. However, QYSX treatment shortened the distance between UC patients and healthy donors (Figure 1(b)). Subsequently, alpha-diversity was also analyzed by gene index, Shannon index, and Simpson index. Gene index showed that after treatment with QYSX, gene number increased compared to that in UC patients without treatment, and the gene number in QYSX group was close to that in normal donors, due to no statistical significance between them (*P* > 0.05, Figure 1(c)). Besides, Shannon index showed that the diversity in UC patients before treatment increased compared to the normal controls and after treatment with QYSX, Shannon index slightly decreased (Figure 1(d)). Simpson index illustrated that there was no significant difference in diversity among the three groups (*P* > 0.05, Figure 1(e)).

**Figure 2:**
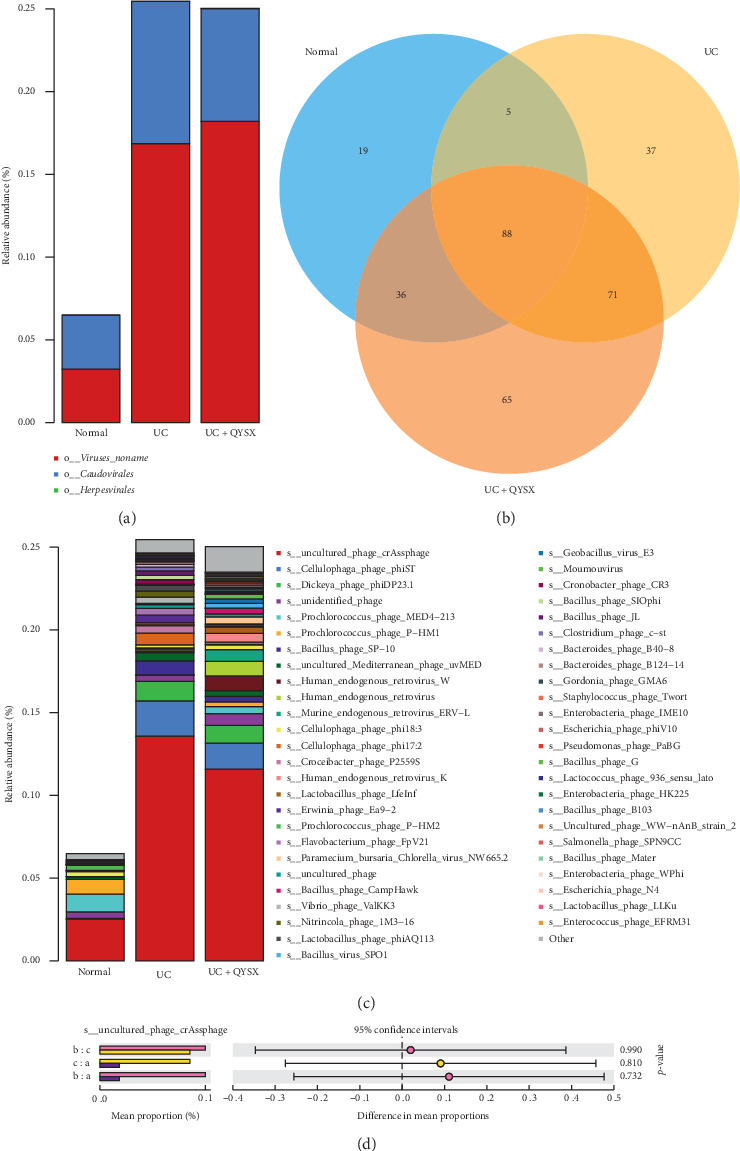
The effects of QYSX on fecal virome composition in UC patients. (a) Relative abundance of fecal virome at order level. (b) Venn diagram analysis of viral species in the three groups. (c) Relative abundance of virome at species level. (d) The diversity of *crAssphage* among the three groups. Note: a, healthy controls; b, UC patients before QYSX treatment; c, UC patients after QYSX treatment.

Based on the sequencing results, 3 main orders of virome were identified, among which *Viruses_noname* was the most dominant in all the three groups, followed by *Caudovirales* and *Herpesvirales* (Figure 2(a)). Moreover, the abundance of *Caudovirales* in UC patients was significantly higher than that in the normal group, and QYSX treatment obviously restored *Caudovirales* abundance in UC patients (*P* < 0.05, Figure 2(a)). Additionally, a total of 321 species of virus were found in this study. Venn diagram analysis revealed that 148 viral species were identified in healthy individuals, and 201 and 260 viral species were found in UC patients before and after QYSX treatment, respectively (Figure 2(b)). Moreover, only 36 species were found in both healthy individuals and QYSX-treated UC patients. A total of 88 viruses were identified in all the three groups, and 19, 37, and 65 viruses uniquely existed in healthy individuals and UC patients before and after QYSX treatment, respectively (Figure 2(b)).

### 3.3. Effects of QYSX on *crAssphage* Diversity of UC Patients

As shown in Figure 2(c), the top 50 viruses were exhibited. Among these top viruses, *crAssphage* was the most dominant species among the three groups. The abundance of *crAssphage* was significantly higher in UC patients (23.5%) than in normal control (2.51%), while the abundance of *crAssphage* was slightly decreased after QYSX treatment, compared with the UC patients (11.59%, Figure 2(c)). Additionally, the mean proportions of *crAssphage* among healthy control group and UC patients before and after QYSX treatment groups were all about 0.1% (Figure 2(d)). There was no significant difference of difference in mean proportions among these groups (*P* > 0.05, Figure 2(d)).

### 3.4. Effects of QYSX on the Other Non-*crAssphage* Bacteriophage Diversity of UC Patients

Based on the results of the top 50 viruses, it is clear that *s__Cellulophaga_phage_phiST* was the second most dominant species, which significantly increased in UC patients (2.13%) compared to healthy individuals (0.009%, Figure 2(c)). In addition, after treatment with QYSX, the relative abundance of *s__Cellulophaga_phage_phiST* (1.57%) significantly decreased in UC patients. Interestingly, *s__Dickeya_phage_phiDP23.1* was found only in UC patients both before and after treatment, suggesting that *s__Dickeya_phage_phiDP23.1* might be harmful in UC. In addition, the abundance of *Bacillus_phage_SP-10* and *Cellulophaga_phage_phi17:2* was also higher in UC patients than in healthy controls, while QYSX therapy effectively reduces their abundance (Figure 2(c)).

Contrary to the trend of the above species, *Prochlorococcus_phage_MED4−213* was the second most enriched species in healthy donators (1.06%), which was significantly reduced in UC patients (0.002%) and elevated in the UC patients after QYSX treatment (0.43%, Figure 2(c)). Moreover, the abundance of *s__Prochlorococcus_phage_P-HM1* in healthy donors, UC patients, and UC patients with QYSX treatment was, respectively, 0.90%, 0.002%, and 0.43%, which suggested that QYSX treatment might increase the abundance of *s__Prochlorococcus_phage_P-HM1* (Figure 2(c)). Similarly, *Prochlorococcus_phage_P-HM2* was also more enriched in healthy controls than in UC patients. Summarily, QYSX may improve the symptoms of UC by altering the abundance of other non-*crAssphage* bacteriophages (*phiST*, *phiDP23.1*, *SP-10*, *phi17:2*, *MED4−213 P-HM1*, and *P-HM2*).

### 3.5. Specific Differences in the Quantity of Viruses before and after Treatment in UC Patients

**Figure 3:**
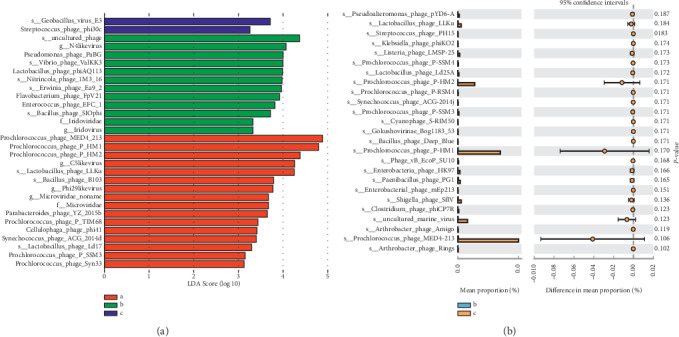
Different fecal virome enrichment in the three groups. (a) Linear discriminant analysis effect size (LEfSe) to identify virus that is differentially represented among healthy donors and UC patients before and after treatment. Note: a, healthy controls; b, UC patients before QYSX treatment; c, UC patients after QYSX treatment. (b) STAMP difference analysis used to compare the abundance of species between before and after treatment in UC patients. Note: b, UC patients before QYSX treatment; c, UC patients after QYSX treatment.

To better understand the differences in the abundance of viruses between before and after treatment in UC patients, linear discriminant analysis effect size (LEfSe) was performed. From Figure 3(a), *Geobacillus_virus_E3* and *phage_phi30c* viruses were enriched in healthy controls, while in UC patients before QYSX treatment, there were 12 enriched viruses observed, including *g__N4likevirus* and *phage_PaBG*. Moreover, in UC patients after QYSX treatment, we found 16 enriched viruses, including *phage_MED4_213*, *phage_P_HM1*, and *phage_P_HM2*. To obtain significant difference in the species treated with QYSX, STAMP difference analysis was used to compare the abundance of the species in UC patients before and after QYSX treatment. As shown in Figure 3(b), *Prochlorococcus_phage_P-HM2*, *Prochlorococcus_phage_P-HM1*, *uncultured_marine_virus*, and *Prochlorococcus_phage_MED4-213* were higher in the UC patients after QYSX treatment, which suggested that these viruses may be beneficial in UC.

### 3.6. Effect of QYSX on Bacterial Composition in UC Patients

**Figure 4:**
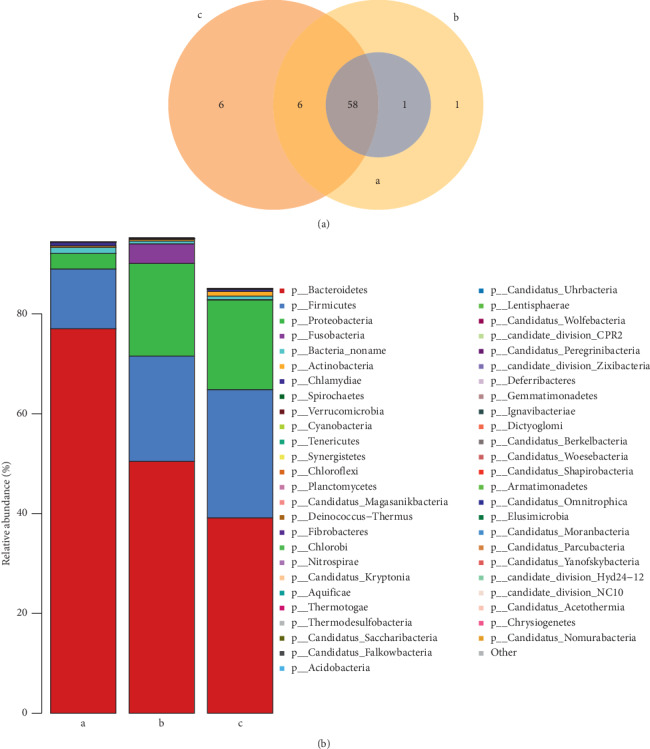
Effect of QYSX on bacterial composition. (a) Venn diagram analysis of the common and unique bacteria at phylum level. (b) Relative abundance of fecal bacteria at phylum level. Note: a, the healthy donors; b, UC patients; c, UC patients after QYSX treatment.

After studying the virome, the changes in the intestinal flora in UC patient were also analyzed. A total of 72 phyla were identified in the three groups. There were 58 phyla present in all the three groups and 6 phyla in UC patients after QYSX treatment. Only one phylum was uniquely identified in UC patients before QYSX treatment (Figure 4(a)). In addition, *Bacteroidetes*, *Firmicutes*, and *Proteobacteria* were the three dominant phyla (Figure 4(b)). Compared to the abundance of *Bacteroidetes*, *Firmicutes*, and *Proteobacteria* in healthy controls, the abundance of *Bacteroidetes* was significantly decreased in UC patients before and after treatment, while the abundance of *Firmicutes* and *Proteobacteria* was significantly increased. Importantly, the abundance of *Fusobacteria* was obviously higher in UC patients before QYSX treatment than in the healthy controls and UC patients after QYSX treatment. This showed that the increase of *Fusobacteria* would possibly lead to the progression of UC.

### 3.7. Different Abundance of Specific Bacteria among All Groups

**Figure 5:**
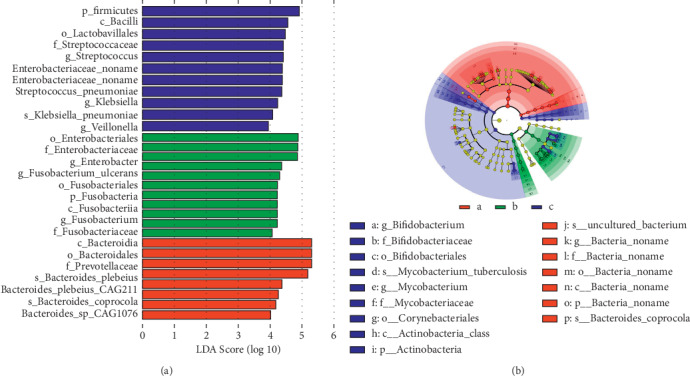
Linear discriminant analysis effect size (LEfSe) to identify differentially enriched bacteria. (a) The bar chart shows differentially enriched bacteria. (b) The circles radiating from the inside out represent the classification levels from the phylum to the genus (or species). Each of small circles at different classification levels represents a classification at that level, and the diameter of the small circle is proportional to the relative abundance. The species with no significant difference were uniformly colored yellow, and the biomarker of the different species followed the group for coloring. Red nodes represent the microbial groups that played an important role in the red group, while green nodes represent the microbial groups that played an important role in the green group, and the color meaning of other circles was similar. Note: blue (a), healthy donors; green (b), UC patients before QYSX treatment; red (c), UC patients after QYSX treatment.

We also performed LEfSe to identify the bacteria that are differentially represented among the three groups. The bar plot displayed that *Firmicutes*, *Bacilli*, and *Lactobacillales* were mainly enriched in healthy controls (Figure 5(a)). The abundance of *Enterobacteriaceae* was higher in UC patients before treatment, while in UC patients after QYSX treatment, *Bacteroidetes* and *Prevotellaceae* were predominant. Additionally, the circle figure showed that in UC patients after QYSX treatment, the abundance of *Bifidobacterium, Mycobacteria* (*Mycobacterium_tuberculosis, Mycobacterium*), *Actinobacteria*, and *Corynebacteriales* were higher, while some types of *Bacteria_noname* and *Bacteroide_coprocola* might play important roles in healthy controls (Figure 5(b)). STAMP analysis showed that 19 species were significantly more enriched in UC patients after treatment than in UC patients before treatment (Figure S2). These species mainly contained *Selenomonas*, *Anoxybacillus*, *Streptococcus*, *Bifidobacterium*, *Bacillus*, *Staphylococcus*, *Lactobacillus*, *Clostridium*, and *Desulfatirhabdium*.

### 3.8. Functional Analysis of Fecal Microflora in the Different Groups

**Figure 6:**
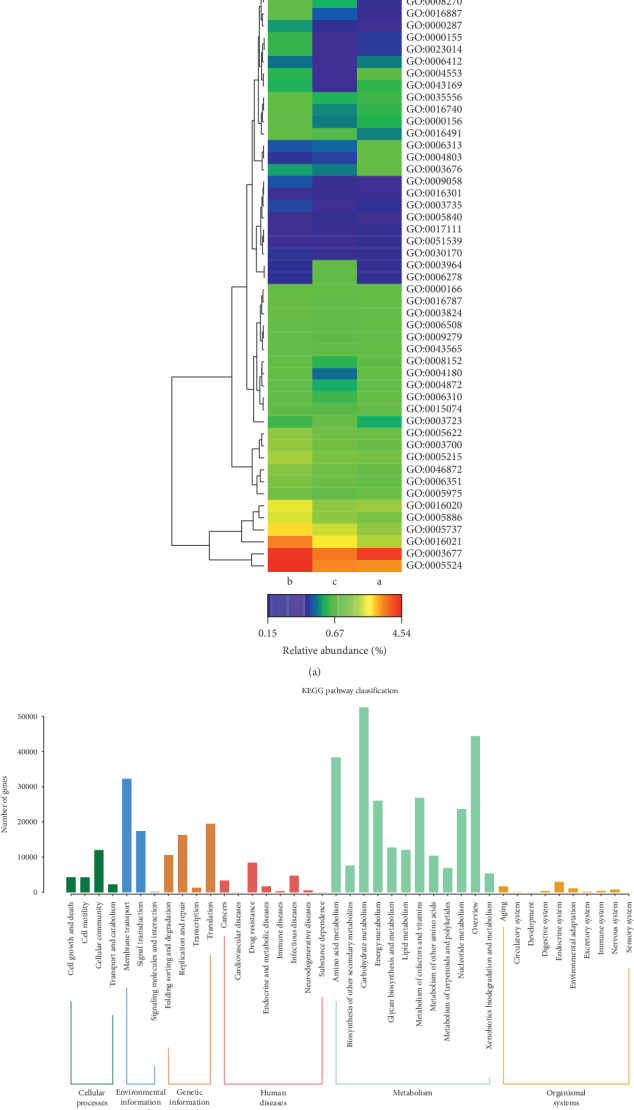
Clustering analysis of Gene Ontology (GO) terms (a) and Kyoto Encyclopedia of Genes and Genomes (KEGG) pathways (b) enriched in fecal microorganism. The red represents high abundance, and blue represents low abundance. Note: a, the healthy donors; b, UC patients; c, UC patients after QYSX treatment.

To explore the potential function of fecal microflora among different groups, GO and KEGG pathway analyses were performed. The results showed that 2778 genes were enriched in all the three groups (Figure S3). Cluster analysis of the functions showed that the functions of microorganism in UC patients after QYSX treatment were similar to those in healthy controls, indicating that the composition of gut microbiome in UC patients after QYSX may be close to that in healthy controls (Figure 6(a)). The functions of regulation of transcription (GO: 0006355), transmembrane transport (GO: 0055085), zinc ion binding (GO: 0008270), ATP binding (GO: 0005524), oxidoreductase activity (GO: 0016491), and ATPase activity (GO: 0016887) were significantly increased in UC patients before treatment compared to healthy controls. After QYSX treatment, these functions decreased compared to those in UC before treatment. Meanwhile, the functions of DNA-mediated transposition (GO: 0006313), transposase activity (GO: 0004803), and nucleic acid binding (GO: 0003676) were decreased in UC patients before treatment. Moreover, KEGG analysis displayed that the identified gut microbiome was mainly involved in cellular processes (cellular community, cell growth, and death), environmental information processing (membrane transport and signal transduction), genetic information processing (translation, replication and repair, folding sorting, and degradation), human diseases (cancers, drug resistance, infectious disease, endocrine, and metabolic disease), metabolism (amino acid, carbohydrate, energy, lipid, nucleotide metabolism, and metabolism of cofactors and vitamins), and organismal systems (endocrine system and environmental adaptation) (Figure 6(b)).

## 4. Discussion

UC is a chronic inflammatory disease that occurs in the gastrointestinal tract, which seriously affects people's health. Presently, morbidity of UC among Asians is increasing due to the improvement in people's standard of living [19]. QYSX, as a traditional Chinese medicine, is widely used to treat many chronic diseases due to its antibacterial, anti-inflammatory, antidiarrheal effects. In our study, QYSX alleviated the damaged colon morphology through endoscopic observation. Subsequently, metagenomic sequencing was applied to study the potential biomarkers of UC and QYSX in 24 fecal samples from healthy individuals, and UC patients before and after QYSX treatment. The results showed that the intestinal microecology in UC patients was disordered, and QYSX altered the composition of gut microorganisms and the functions of the identified microbiota.

In the present study, the diversity of virome increased in UC patients while QYSX slightly decreased the diversity, which is consistent with the previous study [20]. In healthy gut, dsDNA *Caudovirales* and ssDNA *Microviridae* were the characteristic phages [9], while in our study *Caudovirales* and *Herpesvirales* were the dominant order in the three groups. Additionally, the abundance of *Caudovirales* in UC patients was significantly higher than that in healthy individuals. This suggested an abnormal composition of virome in UC patients [21]. However, QYSX significantly decreased the abundance of *Caudovirales* in UC patients compared to UC patients before treatment. Norman et al. [18] showed that gut virome was unbalanced in UC patients, and UC was closely related to a significant expansion of *Caudovirales* bacteriophage.

At the species level, we found that the abundance of *crAssphage* was enhanced in the UC patients compared with the healthy controls, while *crAssphage* diversity was decreased after QYSX treatment. However, there was no significant difference among these groups (*P* > 0.05). It was speculated that this might be due to the relatively small sample size. *CrAssphage*, a newly discovered bacteriophage, is described as the most abundant virus in human gut microbiome [22]. The *crAssphage* similar phages were associated with various bacteria that belong to *Bacteroidetes* [23]. However, a study by Liang et al. reported that *crAssphage* was not related to diarrhea in Chinese patients [24]. Therefore, further research studies on the role of *crAssphage* in UC progression and the effects of QYSX on UC patients should be investigated.

In addition, non-*crAssphage* bacteriophages including *phiST*, *phiDP23.1*, *SP-10*, and *phi17:2* were increased in UC patients, while the abundance of *MED4−213*, *P-HM1*, and *P-HM2* exhibited a trend of decrease. After QYSX treatment, the symptom of UC may be improved by changing the abundance of these viruses. *PhiST* and *phiDP23.1* are two phages of *Cellulophaga*, which is Gram-negative and produce zeaxanthin [25]. *MED4-213*, *P-HM1*, and *P-HM2* are three phages of *Prochlorococcus*, which can serve as organic compounds for the use of nitrogen, phosphorus, energy, or carbon sources [26]. Previous study showed that *Prochlorococcus phages P-SSM2* was significantly associated with black band disease [27]. Although QYSX may affect the composition of gut virome, the specific roles on viruses are still unknown.

Therefore, further analysis was performed to explore the changes and roles of the intestinal flora in different groups. Gut microbiome was proved an essential factor for intestinal inflammation in IBD [28, 29]. In our study, the main phyla of *Bacteroidetes*, *Firmicutes*, and *Proteobacteria* were disordered in UC patients before treatment. Furthermore, the abundance of *Fusobacteria* was obviously higher in UC patients before QYSX treatment than in healthy controls and UC patients after QYSX treatment. Therefore, *Fusobacteria*, a kind of adherent and invasive bacteria, was high in UC patients. A study by Tahara et al. [30] has revealed that *Fusobacterium* is a clinicopathological feature for UC patients in Japan. In addition, the invasive ability of *Fusobacteria* was positively correlated with IBD severity of the host [31–33]. This indicated that *Fusobacteria* may influence the occurrence and progression of UC.

In the present study, LEfSe analysis revealed that *Bifidobacterium*, *Bacteroidetes*, *Prevotellaceae*, *Actinobacteria*, and *Corynebacteriales* were the biomarkers of UC patients after QYSX treatment due to their high abundance. Among them, *Bacteroidetes* related to mucosa have depleted and reduced diversity in patients with IBD [34], and Palmatine can alleviative the symptoms of IBD mice by increasing the abundance of gut *Bacteroidetes* [35]. *Bifidobacterium* can ferment carbohydrates to lactic acid and show an increased tolerance to acidity. Mullner et al. [36] suggested that probiotics such as *Bifidobacterium* might decrease proinflammatory factors (TNF-*α* and IL-10) and increase the expression of anti-inflammatory cytokines (IL-10) by inhibiting the activation of NF-*k*B. *Prevotellaceae*, strictly anaerobic bacteria, has been reported to be high in healthy gut by 16 S rRNA gene pyrosequencing [37], which is in accordance with our study. However, *Mycobacterium*, which comprises of many pathogenic bacteria, was high only in patients treated with QYSX. *Mycobacterium_tuberculosis* is a causative agent for *tuberculosis* [38], which demonstrated that this might be a possible drawback for the use of QYSX. In addition, the roles of *Actinobacteria* and *Corynebacteriales* on UC are still unknown. Therefore, we further explored the functions of these identified gut microbiome.

Based on GO terms of these identified gut microbiome, we found that QYSX could improve UC by mediating regulation of transcription, transmembrane transport, zinc ion binding, ATP binding, oxidoreductase activity, ATPase activity, DNA-mediated transposition, nucleic acid binding, and transposase activity. Among these functions, oxidoreductase activity is closely associated with UC. Some studies have proposed that the imbalance between antioxidant and prooxidant mechanisms may play an important role in the development of intestinal inflammation and mucosal tissue injury in colitis [39, 40]. In addition, KEGG analysis showed that the screened gut microbiome was involved in many pathways such as cell growth and death, genetic information processing, human disease, and various metabolic processes.

However, there are also some limitations in our study. Firstly, the sample size was not large enough. Secondly, this study compared the samples before and after treatment and without a randomized control patient. Besides, the viral particles and bacteria should be further separated from the stool samples to explore the potential molecular mechanism of QYSX in UC.

In conclusion, metagenome sequencing was performed to investigate the composition and function of intestinal microbiota involved in UC and to explore the specific microbiota that responded to QYSX treatment. Our results suggest that the composition and diversity of gut microorganisms may play a key role in mediating UC, highlighting the feasibility of QYSX treatment in UC by modulating intestinal microorganisms.

## Data Availability

The datasets used and analyzed during the current study can be obtained by sending an email to the corresponding author.

## Supplementary Materials

Supplementary Materials Figure S1: analysis of the QYSX effect on UC by endoscopy. Figure S2: STAMP analysis of the fecal bacterial species in the UC patients after treatment compared to the before treatment. Figure S3: Venn diagram shows the unique and the shared GO terms enriched in healthy control (a) and the UC patients before (b) and after (c) QYSX treatment. Table S1: information of UC patients.
