# Supplementary material for: Quyushengxin Formula Causes Differences in Bacterial and Phage Composition in Ulcerative Colitis Patients
Source: Evid Based Complement Alternat Med. 2020 May 11;2020:5859023. doi: 10.1155/2020/5859023 (PMC7240791; doi:10.1155/2020/5859023)
Supplement: Supplementary Materials — Figure S1: analysis of the QYSX effect on UC by endoscopy. Figure S2: STAMP analysis of the fecal bacterial species in the UC patients after treatment compared to the before treatment. Figure S3: Venn diagram shows the unique and the shared GO terms enriched in healthy control (a) and the UC patients before (b) and after (c) QYSX treatment. Table S1: information of UC patients. [file 5859023.f1.pdf]

Supplementary materials

Fig S1 Analysis of the QYSX effect on UC by endoscopy.

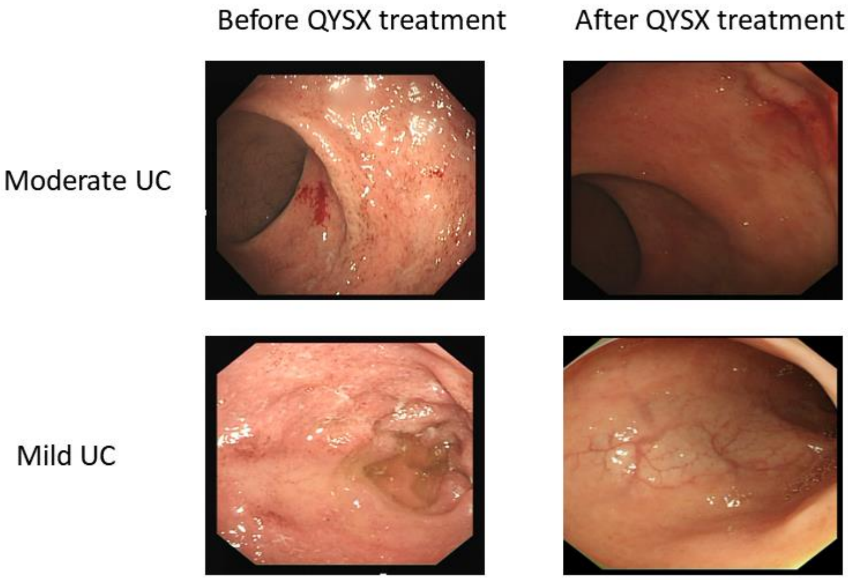

Fig S2 STAMP analysis of gut microbiome in UC patients before and after treatment.

Note: b, UC patients before QYSX treatment; c, UC patients after QYSX treatment.

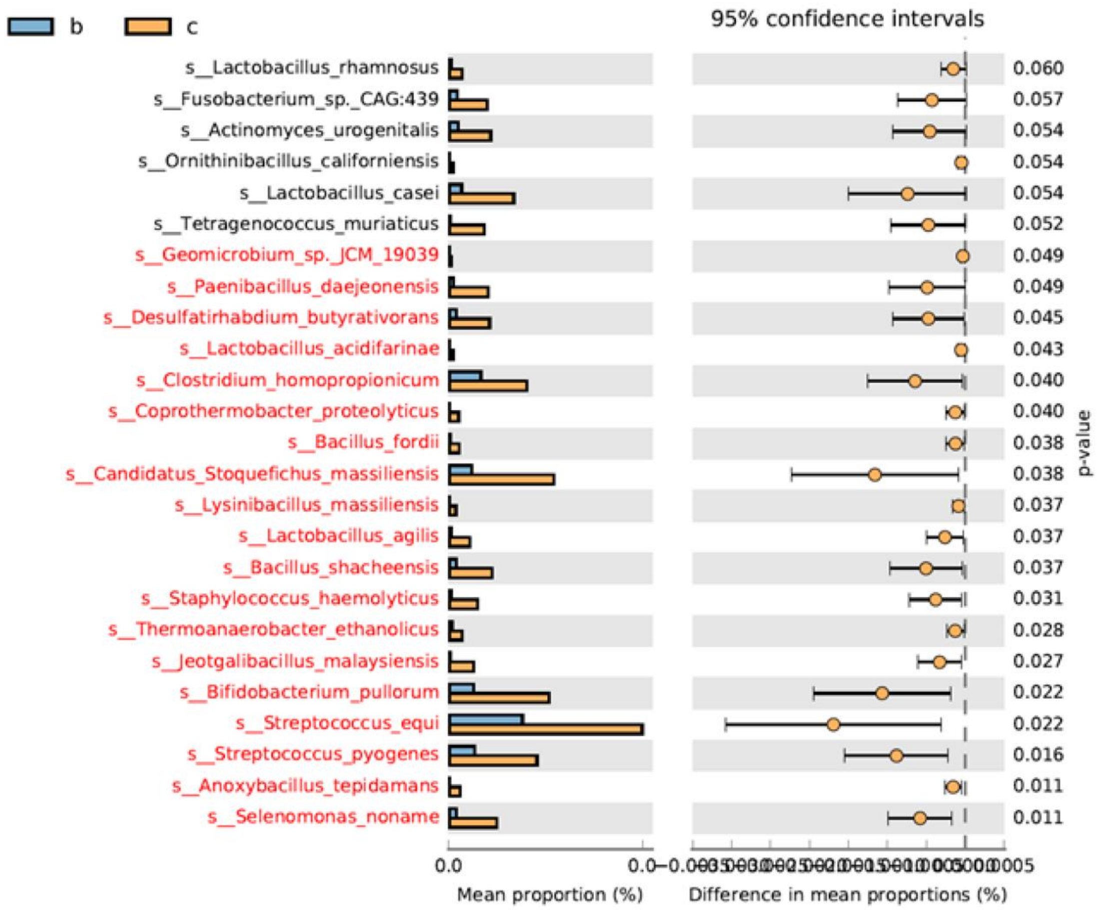

Fig S3 Venn diagram shows the unique and shared GO terms enrich in healthy control (a) and UC patients before (b) and after (c) QYSX treatment.

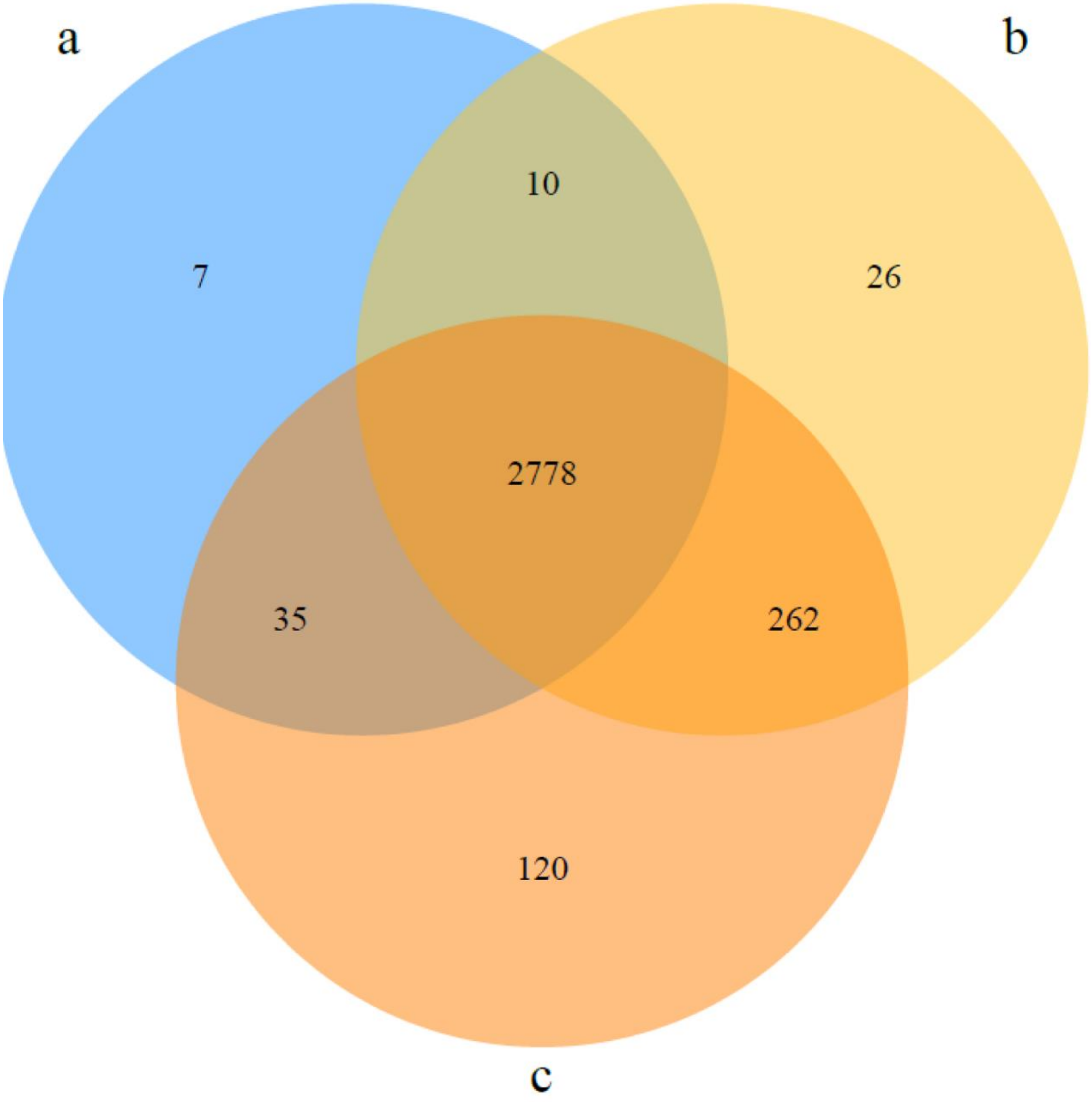

Table S1 Information of UC patients.

Parameters	Classification	Numbers	P value
gender	female	4	
	male	4	---
Montreal classification	E1	3	
	E2	2	---
	E3	3	
Mayo score (before treatment)	5	3	
	7	1	
	8	3	0.0006
	9	1	
Mayo store (after treatment)	3	1	
	4	5	
	5	2	
